# Idiopathic scoliosis in children and adolescents: assessment with a biplanar X-ray device

**DOI:** 10.1007/s13244-014-0354-0

**Published:** 2014-09-13

**Authors:** Elisa Amzallag-Bellenger, Fabian Uyttenhove, Éric Nectoux, Antoine Moraux, Julien Bigot, Bernard Herbaux, Nathalie Boutry

**Affiliations:** 1 Department of Paediatric Radiology, Jeanne de Flandre Hospital, Lille 2 University, University Hospital of Lille, Lille, France; 2 Department of Paediatric Orthopaedics, Jeanne de Flandre Hospital, Lille 2 University, University Hospital of Lille, Lille, France; 3 Service de Radiopédiatrie, Hôpital Jeanne de Flandre, Avenue Eugène Avinée, CHRU de Lille, 59037 Lille, France

**Keywords:** Idiopathic scoliosis, Radiography, Low-dose digital imaging system, 3D reconstruction

## Abstract

Idiopathic scoliosis is one of the most common conditions encountered in paediatric practice. It is a three-dimensional (3D) spinal deformity. Conventional radiography is still the modality of choice for evaluation of children and adolescents with idiopathic scoliosis, but it requires repeat radiographs until skeletal maturity is reached and does not provide information about spinal deformity in all three planes. A biplanar X-ray device is a new technique that enables standing frontal and lateral radiographs of the spine to be obtained at lowered radiation doses. With its specific software, this novel vertical biplanar X-ray unit provides 3D images of the spine and offers the opportunity of visualising the spinal deformity in all three planes. This pictorial review presents our experience with this new imaging system in children and adolescents with idiopathic scoliosis.

• *The biplanar X-ray device produces two orthogonal spine X-ray images in a standing position.*

• *The biplanar X-ray device can assess idiopathic scoliosis with a lower radiation dose.*

• *The biplanar X-ray device provides 3D images of the spine.*

## Introduction

Scoliosis is defined on radiographs by the presence of one or more lateral curvatures of the spine in the coronal plane, greater than 10° as measured by the Cobb method [1]. There are no identifiable causes for this condition in about 80 % of cases and, in particular, no evidence of congenital, developmental or neuromuscular abnormalities [1]. On the basis of patient’s age and clinical features, idiopathic scoliosis is categorised as infantile (0–3 years) scoliosis (male predominance, levoscoliosis more frequent), juvenile (4–10 years) scoliosis (female predominance, dextroscoliosis more frequent) and adolescent (11–18 years) scoliosis (female predominance, dextroscoliosis more frequent) [1].

Radiography is the mainstay to confirm the diagnosis of idiopathic scoliosis in excluding underlying causes (e.g. segmentation abnormalities), to characterise the type of spinal curvature(s), determine the flexibility of the curvature(s), follow disease progression and monitor treatment. Standard evaluation consists of standing frontal radiographs of the entire spine (either anteroposterior [AP] views in males or posteroanterior [PA] views in females in order to reduce the radiation dose to the breasts), sometimes completed by lateral radiographs. However, radiography has two main disadvantages. Firstly, repeated examinations (it is estimated that a typical scoliosis patient will have approximately 22 radiological examinations over a 3-year treatment period [2]) are responsible for an increased amount of radiation exposure and, particularly in young females, an increased risk of breast cancer or infertility [2–5]. Secondly, scoliosis corresponds in reality to a complex three-dimensional (3D) deformity of the spine that simple two-dimensional (2D) radiographs are unable to assess precisely.

During the past decade, a new imaging technique based on George Charpak’s gaseous particle detector technology (Nobel Prize in Physics, 1992) has developed in order to solve these issues [6]. Also known as the EOS imaging system (Biospace Imaging, Paris, France), this digital, biplanar, X-ray imaging acquisition system allows a quick assessment of the entire skeleton in a standing, weight-bearing, position with a significant decrease in radiation dose compared with conventional or other digital radiography systems [7–10]. It also creates 3D images of the skeleton by using computer models [6, 8, 10–15]. The EOS system is therefore particularly well-suited for diagnosis and monitoring of idiopathic scoliosis in children and adolescents [14, 16, 17], as well as leg-length discrepancy and misalignment [9], and for diagnosis and monitoring of degenerative conditions affecting the spine, hips and knees in adults [18]. This pictorial review aims to familiarise radiologists with the EOS imaging system in evaluation of children and adolescents with idiopathic scoliosis.

## EOS 2D/3D system

### EOS 2D system

**Fig. 1:**
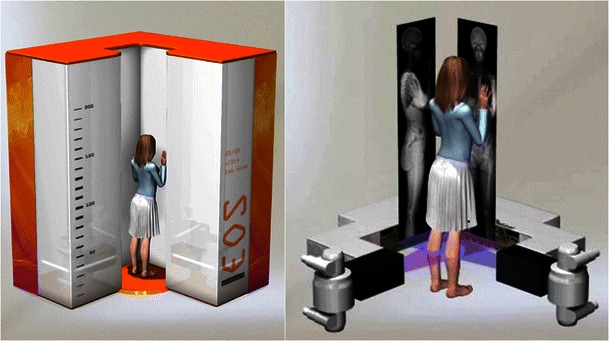
Imaging technique of the EOS 2D system. The gantry is composed of two sets of X-ray tubes and detectors positioned orthogonally and supported by a mobile arm. This arm moves vertically while the patient is positioned upright at the intersection of the two X-ray fan-beams. A single scan can produce both AP and lateral radiographs of the spine, the lower limbs or the whole skeleton

Using two orthogonal sources of radiation and linear detectors that are coupled together, the EOS system simultaneously produces two orthogonal X-ray images of the skeleton in the weight-bearing position. The child or the adolescent is standing upright (or sitting) in the centre of the device (i.e. at the intersection of the two X-ray fan beams) (Fig. 1). Gonadal shielding is usually not applied. Before scanning, the radiology technician defines the limits of the region of interest, in height and width, utilising to two laser beams. The exploration width of the device is limited to 50 cm (corresponding to a lateral diaphragm being wide open). Vertical scanning from head to pelvis for full spine imaging takes about 5–10 s, whereas scanning from head to toe for full body imaging takes about 15–20 s. Only AP or lateral views may also be acquired. If only frontal radiographs are required, a PA projection is used to lessen the radiation dose to the breasts and gonads. Parameters of acquisition (kilovoltage [kV] values, milliampere [mA] values and scanning speed) are variable, depending on the child’s age and weight. The radiology technician can thus choose from three presets: morphotype 1 (slim); 2 (normal) or 3 (corpulent). Acquisition parameters are about 80–90 kV and 200–250 mA for AP views; 100 kV and 250–320 mA for lateral views; scanning speed is chosen between 2 and 4 on the vendor-specific scale (ranging from 1, fast, to 8, slow). In practice, presets 1 and 2 are used for children (age, 5–12 years; weight about 30–45 kg) and adolescents (age, 13–18 years; weight about 45–70 kg) respectively; preset 3 is used only for obese adolescents (weight superior to 75 kg). Like for conventional or other digital radiography systems, child positioning is important to obtain reproducible, comparable radiographs. Among EOS system users, arm positioning is still subject to debate on lateral views because of the superimposition of both humeri on the spine and possible shift in sagittal spinal alignment. The best positioning would be elbows flexed with fists or fingers resting on clavicles or on the cheeks [19, 20]. In our experience, however, this position is not always easy to maintain; at our hospital, when both AP and lateral views are needed, children and adolescents are positioned with the arms supported in front of them, on a bar or on the device wall.

### EOS 3D system

Simultaneous production of two orthogonal X-ray images allows the system to generate 3D images of the skeleton (i.e. the spine, the lower limbs or both) on a dedicated workstation (sterEOS), using key anatomical bony landmarks identified by an operator on the AP and lateral X-ray images, a large statistical database, shape recognition techniques and edge-detection algorithms. The mean reconstruction time is about 20–30 min for 3D spine rendering, 35–45 min for 3D spine and lower limb rendering. It may be much longer (>1 h) for patients with severe idiopathic scoliosis.

## EOS system versus conventional radiography, other digital radiography systems and computed tomography

The EOS system differs from conventional radiography, other digital radiography systems and computed tomography (CT) in several regards: firstly, it allows for imaging in a standing position (or a sitting position with disabled children); secondly, it enables whole-body imaging (if necessary); thirdly, it reduces the radiation dose; fourthly, it creates 3D images of the skeleton.

### Standing position

AP and lateral X-ray images of the spine are acquired in a single vertical scanning mode. Therefore, in contrast to conventional radiography and other digital radiography systems, the EOS system cannot be used in young children with idiopathic infantile scoliosis who cannot stand in the device. In these children, AP and lateral images of the spine have to be performed supine using conventional or, much more frequently, digital radiographic equipment. The latter is now equipped with software that allows automatic stitching of separate cervical, thoracic and lumbar spine radiographs into a single final image. However, some limitations may also be encountered with these techniques (i.e. geometric distortion and stitching errors in conventional and digital imaging respectively) [21].

### Whole-body imaging

**Fig. 2:**
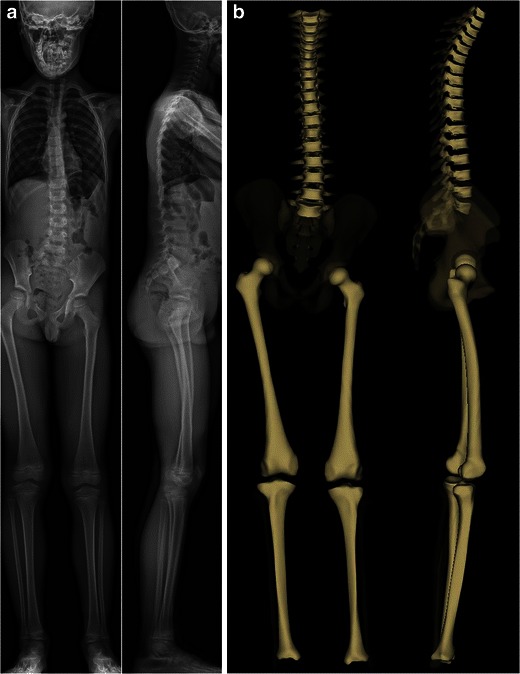
Radiographs of the spine and lower limbs in a 7-year-old boy. AP and lateral images are simultaneously obtained (**a**), allowing for surface 3D reconstructions (**b**). Relationships between the spine and the lower limbs can be assessed. [Total cumulative DAP = 584 mGy·cm^2^]

**Fig. 3:**
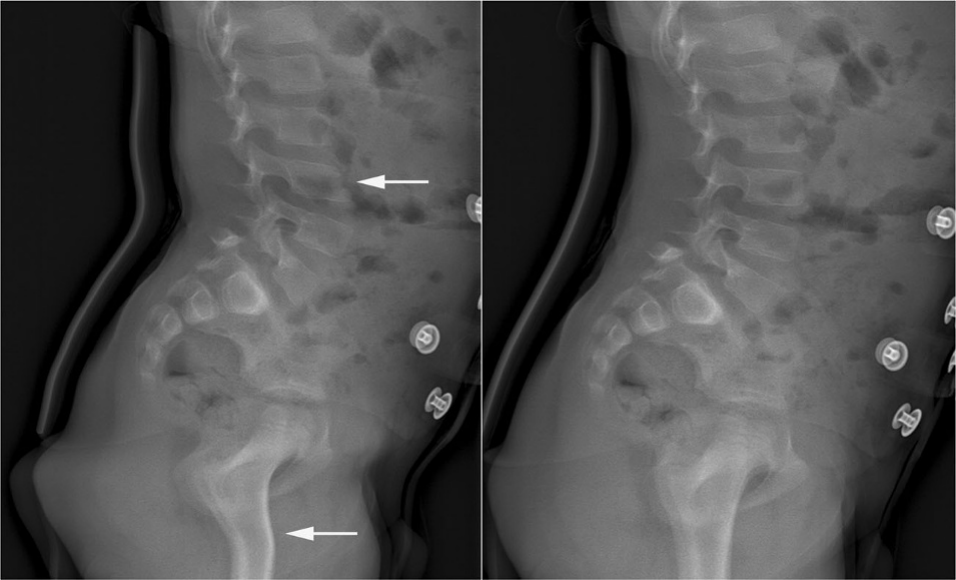
Motion artefacts with the EOS 2D system. Lateral views of the lumbar spine in a 7-year-old boy suffering from an anterior spondylolisthesis of L5 related to a pars interarticularis defect are shown. Child’s movement during the image acquisition (*on the left*) is responsible for a distorted aspect of the spine and the proximal femurs (*arrows*). Compare with another lateral view in the same patient without artefacts (*on the right*). [Total DAP = 140 mGy·cm^2^]

The EOS system can acquire X-ray images of the entire skeleton. This may be very useful for assessing relationships between the spine, the pelvis and the lower extremities in standing functional position (Fig. 2). In fact, significant leg length discrepancy may be responsible for pelvic obliquity and lumbar scoliosis [22]. However, scanning from the base of the skull to the toes requires longer acquisition times; specific artefacts due to patient’s movement therefore may occur in children unable to stay still while performing scanning, resulting in distorted images (Fig. 3).

### Dose reduction

The EOS system allows a significant reduction of radiation dose, by a factor of 2.5–10 compared with 2D conventional radiography and other digital radiography systems [6, 7, 9, 10, 23] and by a factor of up to 800–1,000 compared with 3D CT reconstructions [6]. In our experience, we found a dose reduced by a factor of 4 when comparing our EOS system (average dose area product or DAP of 23.6 mGy·cm^2^/kg ± 4.32) with our digital radiography system (average DAP of 95.7 mGy·cm^2^/kg ± 30.39) in children with idiopathic scoliosis requiring both AP and lateral views.

With the EOS system, the dose depends on kV and mA values, the chosen preset (1, 2 or 3), the scanning speed and the region of interest. For simultaneous AP and lateral radiographs of the spine, the total DAP is around 860 mGy·cm^2^ for morphotype 1 (child), around 1,180 mGy·cm^2^ for morphotype 2 (adolescent) and around 1,780 mGy·cm^2^ for morphotype 3 (obese adolescent). The radiation dose decreases when the translational speed of the X-ray tubes increases (but the image quality decreases as well). It also decreases when the fan-shaped beam of photons is laterally collimated.

### Three-dimensional images of the skeleton

**Fig. 4:**
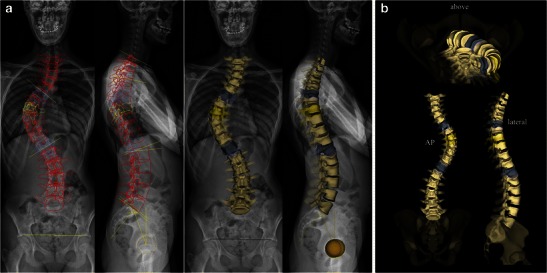
Surface 3D reconstructions of the spine in a 13-year-old girl with idiopathic thoracic scoliosis. A 3D model of the spine (indicated in *red*) is generated by the software and overlayed to the native AP and lateral radiographs (**a**). This model has to be manually adjusted by the operator to precisely match the spinal anatomy. Different perspectives of the spinal deformity are obtained, showing the right thoracic curvature, superior (T6) and inferior (T12) end vertebrae (in *blue*), and the apical vertebra (T8) in *yellow* (**b**). Measurements based on the 3D model are finally automatically computed, including values for spinal curvatures, axial vertebral rotation and pelvic parameters. [Total DAP = 626 mGy·cm^2^]

**Fig. 5:**
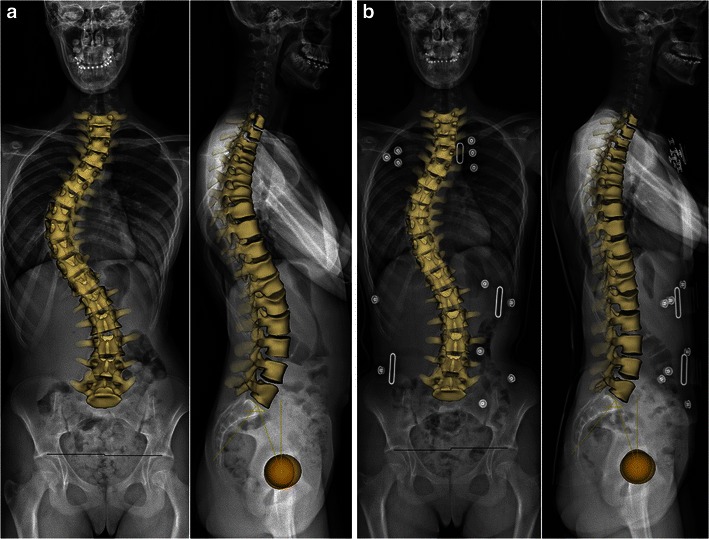
Surface 3D reconstructions of the spine without and with bracing in a 13-year-old girl. Same patient as in Fig. 4, without (**a**) and with (**b**) bracing. [Total DAP without bracing = 626 mGy·cm^2^; with bracing = 1,263 mGy·cm^2^]

**Fig. 6:**
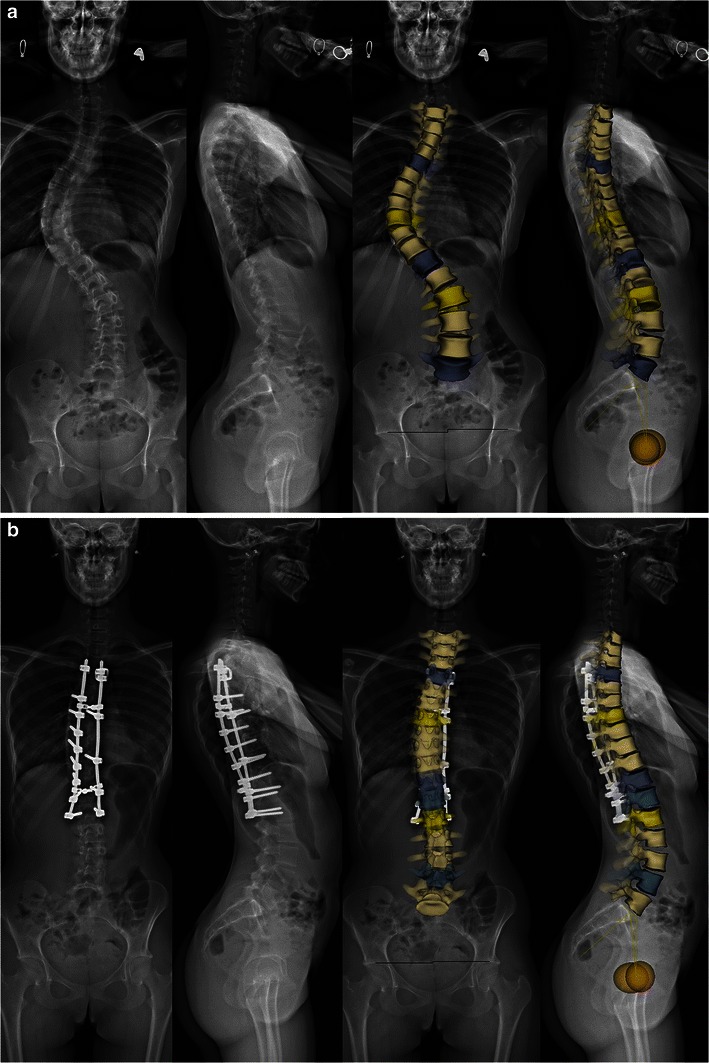
Surface 3D reconstructions of the spine in a 16-year-old girl with idiopathic thoracolumbar scoliosis before and after surgery. There is a major right thoracic curve (apical vertebral T9) and a minor left lumbar curve before surgery (**a**). Significant curve correction is seen after surgery (**b**). Manual adjustment of the 3D model is more difficult after surgery, due to the presence of metal rods, hooks and screws. [Total DAP = 1,868 mGy·cm^2^]

**Fig. 7:**
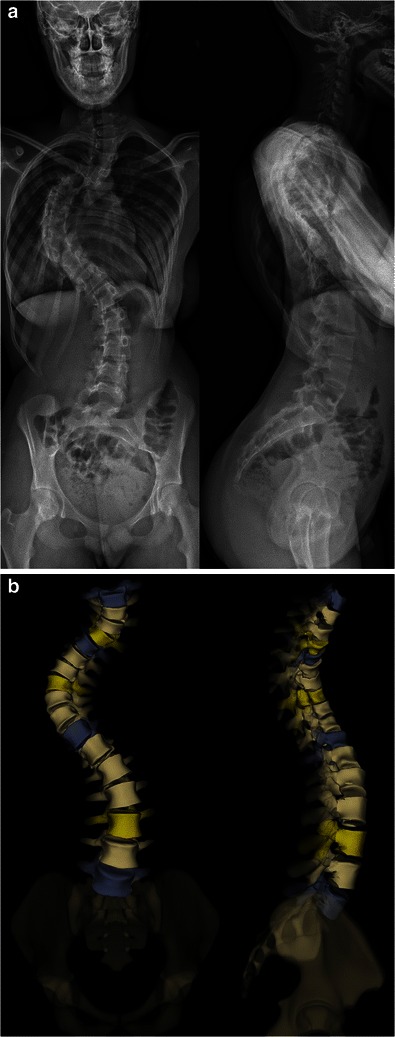
Major right thoracic and left lumbar idiopathic scoliosis in a 14-year-old girl. The severity of the spinal curve on the frontal view and the superimposition of both humeri on the lateral view (**a**) complicate the visibility of the thoracic vertebrae. In this case, the 3D reconstruction process can be very time-consuming. Corresponding surface 3D reconstructions of the spine (**b**). [Total DAP = 812 mGy·cm^2^]

**Fig. 8:**
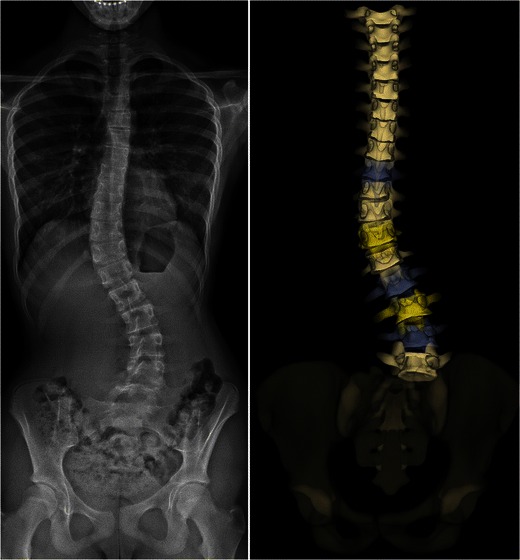
Idiopathic thoracolumbar scoliosis in a 13-year-old girl presenting with a lumbosacral transitional vertebra. The frontal view (*on the left*) shows the presence of six lumbar vertebrae. On the corresponding posterior surface 3D reconstruction of the spine (*on the right*), the last vertebra seems to be dissociated from the pelvis. This is because the pelvis model does not correspond to a true model of the patient’s pelvis. [Total DAP = 1,040 mGy·cm^2^]

Idiopathic scoliosis is characterised by a vertebral deviation in the coronal and sagittal planes but also by a vertebral rotation in the axial (or horizontal) plane. Axial vertebral rotation is difficult to assess on 2D radiographs but it may be explored with CT scans and 3D CT reconstructions [24, 25]. This technique is, however, performed in the supine position; it is limited to short spinal segments and requires a higher radiation dose than conventional or digital radiography, even at low CT doses. In contrast, the EOS system provides large size 3D images of the spine (Fig. 4) from the two lowered-dose X-rays, with no additional radiation and in standing functional position. Three-dimensional EOS images differ from CT reconstructions in that they correspond to surface reconstructions (that are not validated yet in congenital scoliosis) and not real reconstructions. These 3D images provide a better understanding of the spinal deformity from different perspectives (Fig. 4). They may be performed with and without bracing (Fig. 5) or before and after surgery (Fig. 6). Once 3D images are complete, the software automatically generates measurements related to spinal coronal (Cobb angle) and sagittal curves (thoracic kyphosis, lumbar lordosis), and to pelvic parameters. Since they have been computed from 3D space, these measurements have been shown to be more accurate, reliable and reproducible [15, 26]. In current practice, however, some difficulties may be encountered during the 3D reconstruction process. A severe curvature in the coronal plane is responsible for poor visibility of some vertebrae in the sagittal plane, making the adjustment of the model by the operator more difficult (Fig. 7). The presence of lumbosacral transitional vertebrae (i.e. sacralisation of L5 or lumbarisation of S1) is another cause of difficulty since the sterEOS 3D software is not validated yet for this type of anatomical variant (Fig. 8). In this case, the best solution for the operator is to exclude the transitional vertebra from the 3D reconstruction process.

## EOS 2D/3D system in the assessment of idiopathic scoliosis

### EOS 2D system

**Fig. 9:**
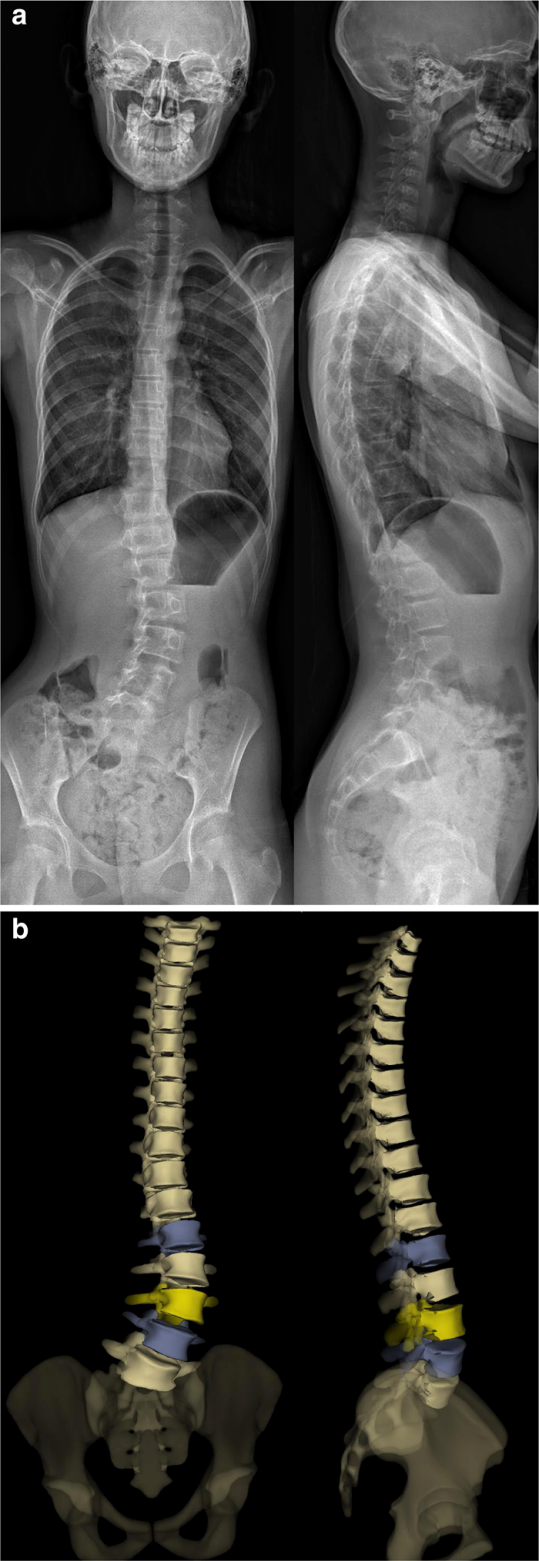
Surface 3D reconstructions of the spine in a 12-year-old girl with lumbar idiopathic scoliosis. AP and lateral images (**a**) and corresponding surface 3D reconstructions (**b**) are shown. [Total DAP = 1,027 mGy·cm^2^]

**Fig. 10:**
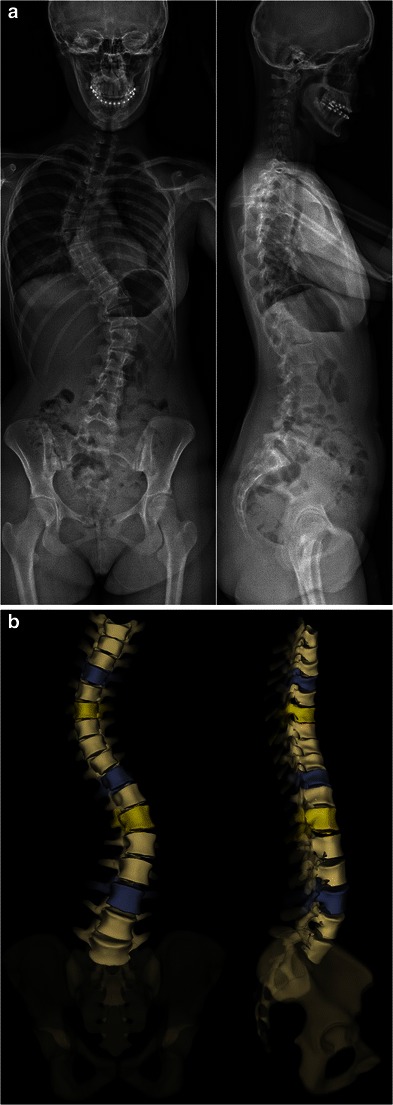
Surface 3D reconstructions of the spine in a 13-year-old girl with double thoracic idiopathic scoliosis. AP and lateral images (**a**) and corresponding surface 3D reconstructions (**b**) are shown. [Total DAP: 1,029 mGy·cm^2^]

**Fig. 11:**
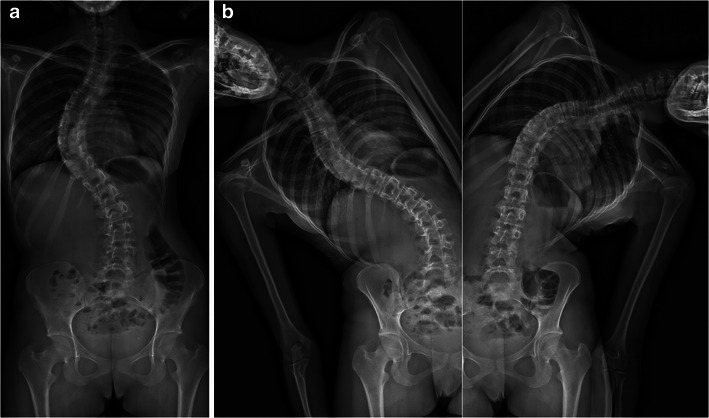
Structural versus non-structural spinal curves in a 15-year-old girl. The AP radiograph reveals right thoracic and left lumbar curvatures (**a**). On bending radiographs (**b**), the right thoracic curve (structural curve) is non-correctable, whereas the left lumbar curve (non-structural curve) is correctable with bending to the left. [Total DAP = 864 mGy·cm^2^]

It can be used to determine the usual spinal and pelvic radiographic parameters in both the coronal and sagittal planes (Table 1), and to assess skeletal maturity [27–34]. According to the Lenke classification system (Table 2), different types of scoliosis may be encountered (Figs. 4, 6, 7, 9 and 10). This classification takes into account the curve type in the coronal plane (structural curve versus non-structural curve[s]), its location (thoracic, lumbar or thoraco-lumbar), its flexibility, and the curves in the sagittal plane to guide surgical treatment of scoliosis [35]. Therefore, it requires standing frontal and lateral spinal radiographs as well as rightward- and leftward-bending radiographs. The latter are useful to make the distinction between the structural curve (also called the primary or the major curve) and the non-structural curves (also called secondary curves or minor curves) before surgery. The structural curve is the largest curve, the one exhibiting more vertebral rotation and the least flexible one (i.e. the one that is non-correctable or partially correctable on ipsilateral sideward-bending with a Cobb angle ≥25°) [35]. It is usually included in operative fusion. In contrast, the non-structural curves are the smallest curves, those exhibiting less vertebral rotation and the most flexible ones (i.e. the ones that are non-correctable or partially correctable on ipsilateral sideward-bending views with a Cobb angle <25°) [1].They develop secondarily, and are usually not included in operative fusion. Rightward- and leftward-bending radiographs are not currently validated in the EOS device, but they may be performed in positioning the patient off-centre within the system (Fig. 11).

**Table 1:** Idiopathic scoliosis: spinal and pelvic radiographic parameters

Coronal Plane	Sagittal Plane
Spinal balance	Spinal balance
Pelvic obliquity	Spinal parameters
	- Thoracic kyphosis
	- Lumbar lordosis
Scoliosis	Pelvic parameters
- Type and side of spinal curve(s)	- Sacral slope
- Cobb angle	- Pelvic tilt (or pelvic version)
- End vertebrae	- Pelvic incidence
Severity of axial rotation (apical vertebra)	
Risser index

**Table 2:** Lenke classification system

Curve type		Lumbar spine modifier^a^	Sagittal thoracic modifier^b^
Type 1	main thoracic	A	–
Type 2	double thoracic		
Type 3	double major	B	N
Type 4	triple major		
Type 5	thoracolumbar/lumbar	C	+
Type 6	thoracolumbar/lumbar main thoracic		

^a^Lumbar spine modifier is assigned on the basis of the relation between the lumbar apical vertebra and the centre sacral vertical line (CSVL): *A* if the CSVL lies between the pedicles of the apical vertebra; *B* if the CSVL touches the apical vertebral body and *C* if the CSVL lies completely medial to the apical vertebral body

^b^Sagittal thoracic modifier is assigned on the basis of the sagittal alignment from T5 to T12: – if the angle of kyphosis is less than 10°; *N* if the angle of kyphosis is 10–40°; + if the angle of kyphosis is greater than 40°

### EOS 3D system

**Fig. 12:**
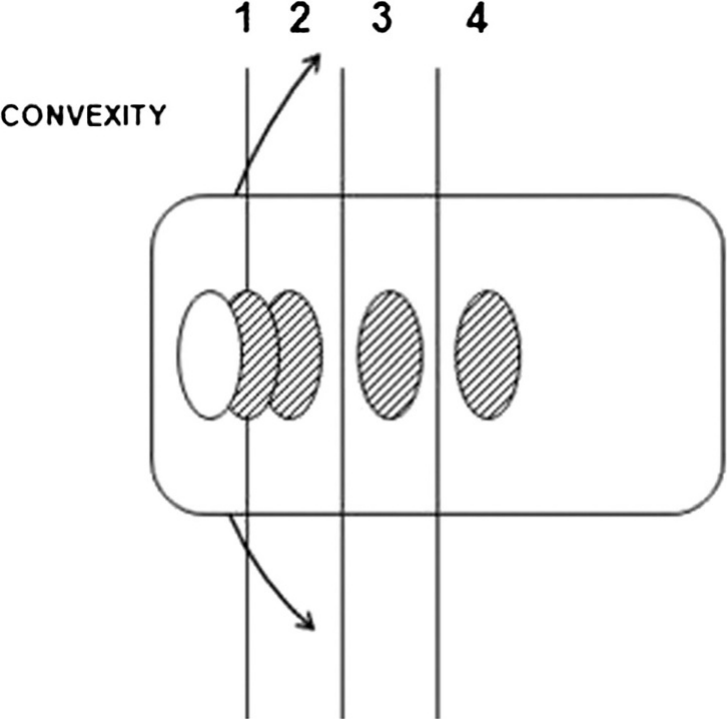
Axial vertebral rotation assessed by the Nash-Moe method. This 2D method uses the position of pedicles as reference landmarks. The half vertebra on the side of convexity is divided into three segments. If there is no axial vertebral rotation (grade 0), the pedicle is seen within the outer segment. As the degree of axial vertebral rotation increases (grades 1–4), the convex-side pedicle migrates towards the side of concavity

**Fig. 13:**
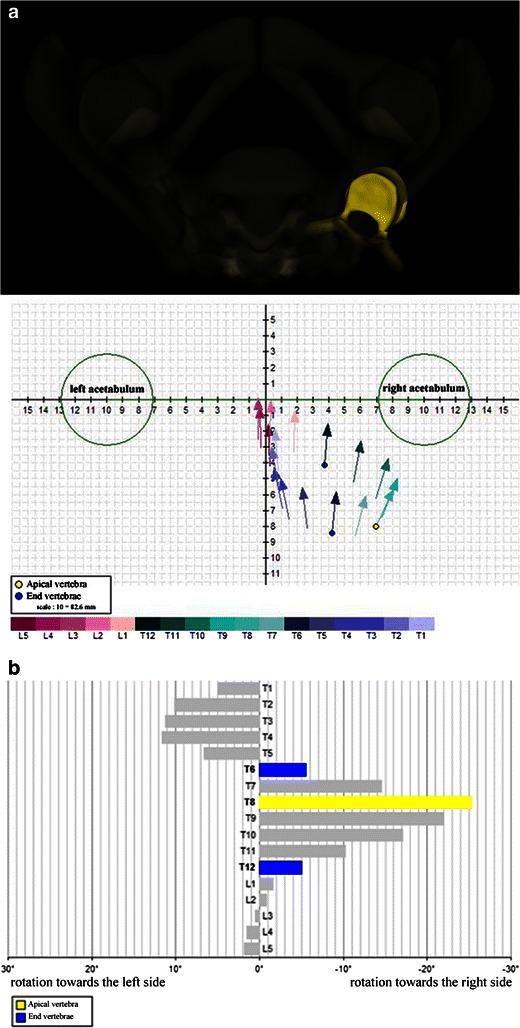
Axial vertebral rotation assessed by the EOS method. This 3D method shows position and rotation of the apical vertebra (in *yellow*) on a top view (**a**). With this method, each vertebra is represented by a vector, which is placed in a coordinate system (*x*, *y* and *z* where *x* corresponds to the interacetabular axis). This vertebral vector gives information about position and rotation of the vertebra in horizontal plane (note: the scale is based on the interacetabular distance). Apical (*yellow*) and end (*dark blue*) vertebrae are indicated (**a**). Another way of showing position and rotation of apical (T8) and end (T6, T12) vertebrae in horizontal plane is given by a diagram (**b**)

**Fig. 14:**
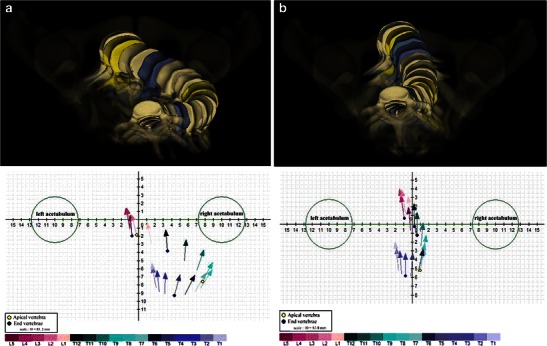
Axial vertebral rotation assessed by the EOS method before and after surgery. Corresponding top view 3D reconstructions and vertebral vectors, before (**a**) and after (**b**) surgery in the same patient as in Fig. 6

It can be used to measure the degree of axial vertebral rotation. This is usually assessed semi-quantitatively on frontal radiographs by different methods, in which the spinous process location (Cobb) [36] or the pedicle location [37–39] are used as indirect indicators of the severity of axial vertebral rotation. In our institution, we prefer the Nash-Moe method (Fig. 12) or each time if possible, the direct assessment of axial vertebral rotation with the EOS system and its top view method (Fig. 13). This method shows the position and rotation of the apical vertebra in the horizontal plane based on the interacetabular distance (Fig. 13). More recently, via the concept of “vertebral vectors”, Illés et al. [17, 40] found another way to visualise the position of all vertebrae, including the apical one, in the horizontal plane and, most importantly, to quantify the vertebral rotation in all three planes simultaneously (Fig. 13). This may be used to show the evolution of scoliosis before and after surgery (Fig. 14).

In conclusion, radiography plays a pivotal role in the evaluation of children and adolescents with idiopathic scoliosis. However, it is limited to 2D measurements of frontal and sagittal spinal curves, and regular follow-up until skeletal maturity requires repeated X-ray exposure. The EOS 2D/3D system is a biplanar X-ray system that appeared in 2005 to overcome these drawbacks. It allows imaging of the spine at lowered radiation levels. Another advantage is the possibility of obtaining 3D images of the spine in the standing functional position. This new imaging technique is therefore increasingly being used in paediatric imaging departments. In the present article we have provided an overview of the potential usefulness of the EOS 2D/3D system in children and adolescents with idiopathic scoliosis; however, it appears too early to assess precisely its 3D ability and its impact on therapeutic management.
